# Current State of Therapeutics for HTLV-1

**DOI:** 10.3390/v16101616

**Published:** 2024-10-15

**Authors:** Tiana T. Wang, Ashley Hirons, Marcel Doerflinger, Kevin V. Morris, Scott Ledger, Damian F. J. Purcell, Anthony D. Kelleher, Chantelle L. Ahlenstiel

**Affiliations:** 1 Kirby Institute, University of New South Wales, Sydney, NSW 2052, Australia; twang@kirby.unsw.edu.au (T.T.W.); sledger@kirby.unsw.edu.au (S.L.); akelleher@kirby.unsw.edu.au (A.D.K.); 2 The Peter Doherty Institute for Infection and Immunity, University of Melbourne, Melbourne, VIC 3052, Australia; ashley.hirons@unimelb.edu.a (A.H.); dfjp@unimelb.edu.au (D.F.J.P.); 3 Walter and Eliza Hall Institute of Medical Research, Parkville, VIC 3052, Australia; doerflinger.m@wehi.edu.au; 4 Department of Medical Biology, University of Melbourne, Melbourne, VIC 3050, Australia; 5 Centre for Genomics and Personalised Health, School of Biomedical Sciences, Queensland University of Technology, Kelvin Grove, QLD 4059, Australia; kevin.morris@qut.edu.au; 6 UNSW RNA Institute, University of New South Wales, Sydney, NSW 2052, Australia

**Keywords:** retrovirus, HTLV-1, antiviral therapeutics, ATL, HAM

## Abstract

Human T cell leukaemia virus type-1 (HTLV-1) is an oncogenic retrovirus that causes lifelong infection in ~5–10 million individuals globally. It is endemic to certain First Nations populations of Northern and Central Australia, Japan, South and Central America, Africa, and the Caribbean region. HTLV-1 preferentially infects CD4^+^ T cells and remains in a state of reduced transcription, often being asymptomatic in the beginning of infection, with symptoms developing later in life. HTLV-1 infection is implicated in the development of adult T cell leukaemia/lymphoma (ATL) and HTLV-1-associated myelopathies (HAM), amongst other immune-related disorders. With no preventive or curative interventions, infected individuals have limited treatment options, most of which manage symptoms. The clinical burden and lack of treatment options directs the need for alternative treatment strategies for HTLV-1 infection. Recent advances have been made in the development of RNA-based antiviral therapeutics for Human Immunodeficiency Virus Type-1 (HIV-1), an analogous retrovirus that shares modes of transmission with HTLV-1. This review highlights past and ongoing efforts in the development of HTLV-1 therapeutics and vaccines, with a focus on the potential for gene therapy as a new treatment modality in light of its successes in HIV-1, as well as animal models that may help the advancement of novel antiviral and anticancer interventions.

## 1. Introduction

Detected in 1980 [1,2] through the isolation of T-lymphoblastoid cell lines, human T cell leukaemia virus type-1 (HTLV-1) became the first discovered oncogenic retrovirus, following the characterisation of reverse transcriptase in 1970 [3,4]. HTLV-1 is a type of Deltaretrovirus, belonging to the Orthoretrovirinae subfamily, and causes life-long infection [5] in an estimated 10 million individuals worldwide across a vast geographical area, with endemic hotspots vastly limited to Indigenous populations [6,7,8,9]. HTLV-1 was first identified as the aetiologic agent of adult T cell lymphoma/leukaemia (ATL) [10], with the prevalence of 5% amongst carriers [11,12]. Since its discovery, further characterisation has suggested that HTLV-1 carries a much broader burden of disease globally, being implicated in a range of immune-associated diseases and systemic inflammation, amongst other comorbidities [13,14,15,16,17].

### 1.1. Epidemiology

There are many obstacles facing the accurate estimation of HTLV-1 global infections and seroprevalence due to the absence of population-based studies and inconsistencies in global diagnostic strategies. The global prevalence of HTLV-1 has been estimated to be anywhere between 3 and 30 million cases [18,19], with estimates based primarily on the serological screening of blood donors, pregnant women, and at-risk population groups [9,20,21,22]. Whilst HTLV-1 infection has been documented in every continent, its endemicity clusters at focal points in regions where the virus is nearly absent. In these focal points, the seroprevalence of HTLV-1 in adults ranges from 0.1% to up to 40%, increasing with age and in people assigned female at birth [23,24,25,26]. 

**Figure 1:**
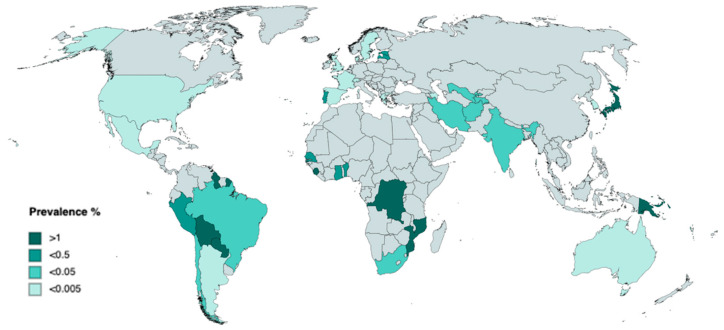
Worldwide distribution of HTLV-1 infection. The map specifies the prevalence by region. Created with mapchart.net (2022).

Due to its low sequence variation, phylogenetic studies can be used to trace the origins of HTLV-1 to Central Africa, with Africa being the only continent where all different forms of the Primate T-Lymphotropic Virus (PTLV) can be found [21,27]. PTLV consists of three genotypes of Human T cell Leukaemic retroviruses (HTLV-1, 2, and 4) and four non-human primate Simian T cell Leukaemic retroviruses (STLV-1, 2, 3, and 4); however, only HTLV-1 has been linked to disease [28]. The molecular genotypes of HTLV-1 can be grouped into seven subtypes, which are determined by the few nucleotide substitutions within the proviral genome of each strain. The remarkable genetic stability of HTLV-1 can be attributed to its preferred mode of amplification, clonal expansion, thus bypassing the nucleotide mutations frequently introduced by the error-prone reverse transcriptase. These subtypes are as follows: cosmopolitan (subtype A), Central Africa (Subtype B), Australo-Melanesian (Subtype C), Central African Republic (Subtype D), and a limited number of strains in Central Africa (Subtypes E, F, G) [26]. Cosmopolitan subtype A is present across multiple endemic clusters, including southern Japan, the Middle East, South America, and South Africa (Figure 1). 

### 1.2. HTLV-1 Genome

While HTLV-1 is a genetically complex and robust retrovirus, it has a relatively small 9 kB single-stranded(+) RNA genome. The genome itself contains structural and regulatory genes, flanked by two identical long terminal repeats (LTRs) at the 5′ and 3′ region. The LTR is further subdivided into three regions (U3, R, U5), which contain the cis-acting elements required for the expression of viral genes (Table 1). Following infection, HTLV-1 is integrated into the host cell chromatin, and the LTRs act as promoters for both the sense and anti-sense strand directions [29], expressing multiple gene products detailed in Table 1. 

**Table 1:** A summary of HTLV-1 genes, protein products, and functions.

Gene	Protein	Function	Localisation
*Gag*	MA	Matrix [30]	Virion [31]
	CA	Capsid [30]	Virion [31]
	NC	Nucleocapsid [30]	Virion [31]
*Pro*	PR	Protease; cleavage of Gag and Pol [32]	Virion [32]
*Pol*	RT	Reverse Transcriptase; reverse transcription of HTLV-1 RNA into DNA [30]	Virion [33]
	RH	RT-RNase H; mediates RNA cleavage during replication and repair [30]	Virion [33]
	IN	Integrase; DNA provirus integration [30]	Virion [33]
*Env*	SU	Surface subunits bind to host cell surface receptors to facilitate fusion of viral and host cell membranes [30]	Plasma membrane [34]
	TM	Transmembrane proteins [30]	Plasma membrane [34]
*Rex*	Rex (p27)	RNA-binding post-transcriptional regulator; promotes export of spliced viral RNA from the nucleus to the cytoplasm [35]	Primarily in the nucleoli/nucleus exported to the cytoplasm [35]
*Tax*	Tax (p40)	Activates viral transcription by interacting with enhancer elements in the 5′LTR and activating CREB/ATF, NF-κB and AP1 pathways [36]	Primarily in the nucleoli/nucleus exported to the cytoplasm [37]
*HBZ*	HBZ	Negative regulator of TAX-mediated transcription. Expression of HBZ mRNA stimulates lymphocyte proliferation [38]	Nucleus/cytoplasm [39,40]
*p12*	p12	Multiple functions to promote escape of immune surveillance and T cell proliferation [41]	Endoplasmic Reticulum [42]
*p13*	p13	A truncated form of p30 which alters the membrane potential and reactive oxygen species (ROS) production [43]	Mitochondria [42]
*p30*	p30	Inhibits viral expression and promotes repression by regulating *tax*/*rex* mRNA [44]	Nucleus [45]

**Table 2:** Transcriptional activators of HTLV-1.

Name	Sequence
NF-*κ*B/NFAT [46]	AA…GGGGCTCCT…CA
NF-YB/CBEP*β* [47]	GG…CCAAT…GT
Sp1/Sp3 [48]	AA…CCACCC…AT
TRE-1 repeat I (vCRE-1) [49]	AGGC…TGACGTCT…CCCC
TRE-1 repeat II (vCRE-2) [49]	AGGC…TGACGTGT…CCCC
TRE-1 repeat III (vCRE-3) [49]	AGGC…TGACGACA…CCCC
TF-IIA/TF-II D [50]	TC…TATAA…AA
Elk-1/SRF [51]	CCGGGAA…CCGGGAA…CCATGTTTGT
AP-1 [52]	CA…TGAG…CC

Transcription factor binding sites have been underlined. Tax is a major transcriptional activator of HTLV-1. Tax1 mediates majority of transcriptional activation via interactions with transcription factors, including the basal transcription factors TF-IIA/D, as well as AP-1, Sp-1/Sp3, and SRF/Elk-1. Additionally, Tax activates nuclear factors NF-κB and NF-YB and forms a complex with ATF/CREB members on vCREs (1–3) by binding to the A and C domains to mediate transactivation.

**Figure 2:**
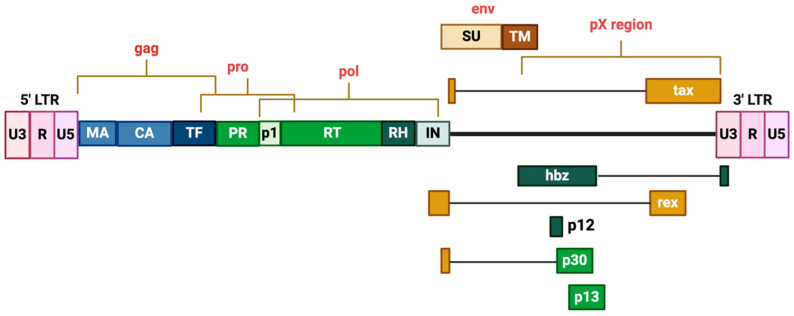
The genomic structure of HTLV-1. The 3′ region is described as the pX region. Tax, rex, and hbz are spliced. Created in BioRender.

The genome encodes for essential retroviral structural proteins and enzymes including Gag, Pro, Pol, and Env, as well as regulatory and accessory proteins at the 3′ region (Figure 2). Before the 3′LTR lies the pX region, where alternatively spliced mRNAs from positive and negative RNA strands encode for functional proteins [53,54], many of which act as transcriptional activators and are critical for disease pathogenesis by facilitating a balance between the proliferation and persistence of infected cells [55,56]. Table 2 present here are also two regulatory proteins, Tax and Rex, which are trans-activators of transcription, the inhibition of which by the HTLV-1 p30II viral protein inhibits virus expression and suppresses Tax/HBZ-induced oxidative and metabolic toxicity [57,58]. Recently discovered within the pX region lies the HTLV-1 basic domain/leucine zipper factor (HBZ), an oncoprotein that modulates cellular pathways to negatively regulate Tax-dependent proviral gene transcription [57,58,59,60]. 

### 1.3. HTLV-1 Transmission and Life Cycle

#### 1.3.1. Transmission

HTLV-1 mostly requires cell-to-cell contact for effective transmission [61], with de novo cell-to-cell transmission and contact with bodily fluids containing infected cells being the most common. There are three modes of transmission between hosts: parenteral transmission (blood and blood products), vertical transmission (during breastfeeding or parturition), and sexual transmission [62]. The most common mode of transmission is breastfeeding [63], with a high proviral load (PVL) in breast milk [64] and high serum HTLV-1 antibody titres. However, the precise mechanisms of HTLV-1 transmission through breastfeeding and sexual contact remain unclear, given that the virus is primarily transmitted through cell-to-cell contact of T cells, and further investigation into this process is warranted. By nature, retroviruses are more genetically unstable due to sequence variations introduced by reverse transcriptase. However, the genetic stability of HTLV-1 can be attributed towards its preferred method of viral amplification: the clonal expansion of infected cells. Cell-to-cell propagation is favoured above cell-free de novo infection and is facilitated by three mechanisms [65]: viral synaptic transfer, extracellular biofilms [66], and mitosis of cells containing integrated provirus. Cell polarisation occurs upon contact with a target cell to facilitate a viral synaptic transfer. Gag, Env, and genomic RNA proteins localise towards the viral synapse [67] to cause a rapid onset of transmission through budding and fusion of the HTLV-1 virion [68,69,70]. In addition to transmission through the viral synapse, HTLV-1-infected cells can form extracellular biofilm-like structures composed of carbohydrates and linker proteins [66], which increases the likelihood of infection of a permissive target cell [71]. 

#### 1.3.2. Life Cycle

HTLV-1 primarily infects CD4^+^ T cells, although it has the capacity to infect a wide variety of cells due to its widely distributed cell surface receptors—glucose transporter (GLUT-1), heparin sulphate proteoglycan (HSPG), and the vascular endothelial growth factor-165 receptor neuropilin-1 (NRP-1) [72]. This includes the infection of CD8^+^ T cells, endothelial cells, myeloid cells, fibroblasts, and other mammalian cells [73]. 

**Figure 3:**
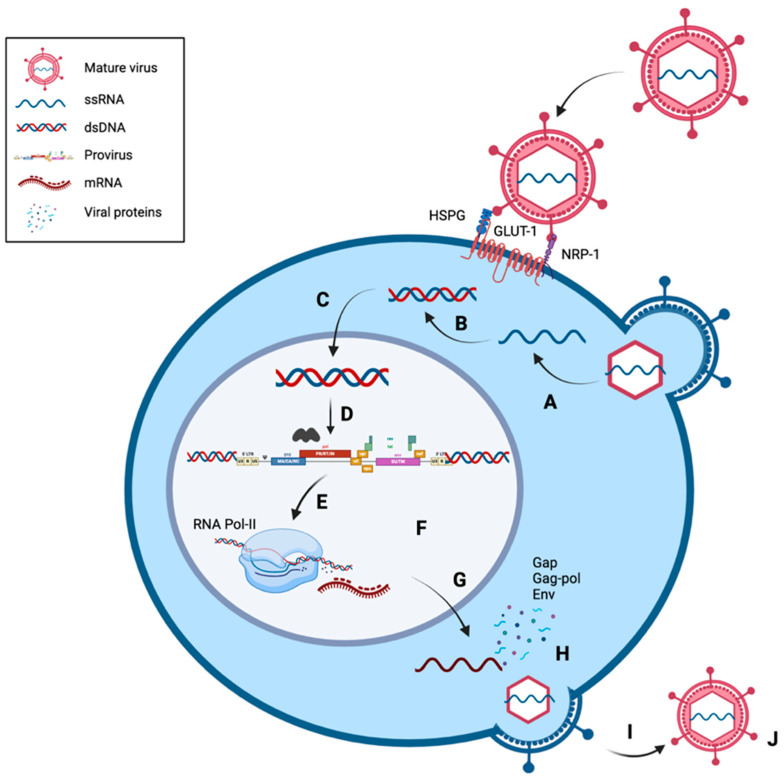
The HTLV-1 life cycle in a cell. The virus fuses into the host cell, CD4^+^/CD8^+^ T cells, through interaction with cell surface receptors. The viral core is transported into the cytoplasm where it is reverse transcribed into a double-stranded DNA. This is then transported into the nucleus and integrated into the host genome. The viral proteins are transcribed and translated by the host cell machinery and become infectious after undergoing viral budding. Created in BioRender.

The HTLV-1 envelope protein is first synthesised as a precursor gp61 protein, before cleavage into its gp46 (the surface subunit, SU) and gp21 (the transmembrane, TM) counterparts. Similar to HIV-1, SU and TM are theorised to be crucial to viral entry. SU then interacts with HSPG and then NRP-1 to form the HSPG/NRP-1 complex, which then associates with GLUT-1 to initiate the fusion process [74]. The fusion itself is receptor mediated, through interactions with the HTLV-1 Env TM proteins. This allows the HTLV-1 capsid core, which contains the viral genomic RNA and enzymes such as reverse transcriptase, integrase, and viral protease, to be released into the cytoplasm of the target cell [75] (Figure 3 (A)). Upon entry, single-stranded RNA undergoes reverse transcription by reverse transcriptase (RT) to convert the genome into double-stranded DNA (Figure 3 (B,C)). The double-stranded viral DNA is then transported into the nucleus and incorporated into the host genome to form the integrated provirus via integrase (IN) (Figure 3 (D,E)). The provirus, acting as the template for viral RNA production, is then transcribed by RNA polymerase II, post transcriptionally modified, and exported from the nucleus into the cytoplasm (Figure 3 (F,G)). In the cytoplasm, they are translated by the host cell to form an RNA genome. This is assembled along with Gag, Gag-pol, and Env proteins along the plasma membrane to a virus budding site to form an immature virus particle (Figure 3 (H)). The particles are released from the cell surface to undergo maturation by cleavage through the viral protease, forming an infectious and mature viral particle (Figure 3 (I,J)). 

### 1.4. HTLV-1 Persistence

HTLV-1 infection expands in the host largely through the proliferation of infected cells undergoing mitosis. Existing primarily as a stable provirus in vivo, HTLV-1 can theoretically persist indefinitely without the need for transcription. The majority of infected cells exist as memory CD4^+^ T cells, allowing for long-term persistence [76,77,78,79], and the subsequent transcriptional silence serves as a potent mechanism evasion of immune system detection. The retrovirus maintains a robust system of regulating re-expression using the two genes *tax* and *hbz*, primarily for host-to-host transmission, cellular immortalisation, proliferation, and pathogenesis. Transcriptional activity is regulated at the epigenetic level through changes in the 5′ viral promoter in the LTR, namely via DNA methylation or at the genetic level by deletion. DNA methylation of the 5′LTR silences transcription from the sense strand and is highly selective and potent in nature [80,81]. The deletion of the 5′LTR is observed in malignant clones of ATL in 30% of cases, approximately half of which was deleted during clinical latency [82,83]. *Tax* mRNA was found to be expressed in only 40% of ATL patients, whereas *hbz* is uniformly expressed in ATL and HAM cells [39,84,85,86], suggesting that it plays an indispensable role in the maintenance of leukaemia and other HTLV-1-associated diseases [87]. Despite the increasingly better characterisation of the pathogenesis of these diseases, the long period of clinical latency remains poorly understood. Many comparisons can be drawn between the latency of HTLV-1 and that of HIV-1, primarily as the two viremias persist asymptomatically by residing silently in memory CD4^+^ T cells of the periphery [88,89]. When untreated, HIV-1 persists through infected CD4^+^ T cells undergoing active replication, rarely presenting in forms of true transcriptional latency [90]. By contrast, HTLV-1-infected CD4^+^ T cells survive and proliferate indefinitely through clonal expansion, with low levels of ongoing transcription and bursts of transcriptional activity (Table 3).

### 1.5. HTLV-1-Associated Diseases

**Figure 4:**
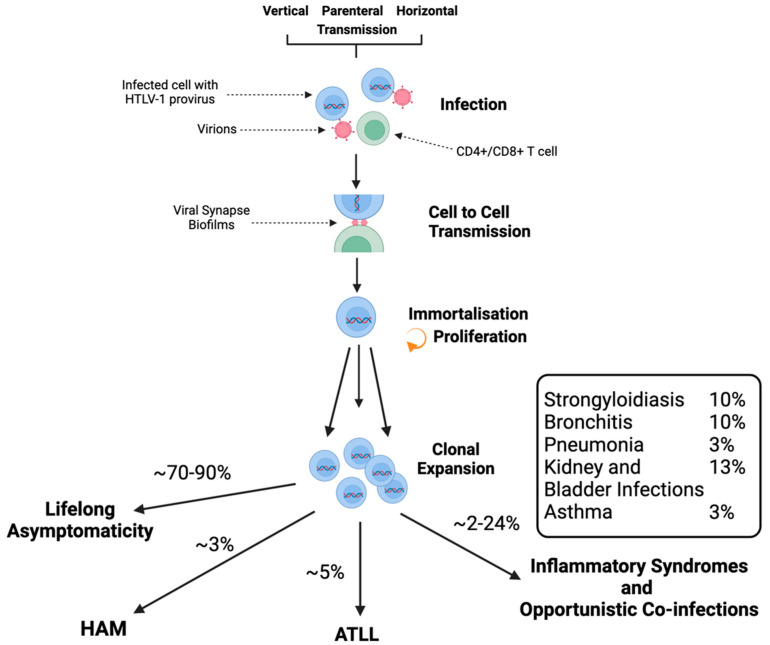
Disease progression of HTLV-1 and the rates of infection. HTLV-1 mostly presents in infected patients as a lifelong subtle immune dysfunction without a defined associated disease, with a broadly estimated 10–30% of populations developing symptomatic HTLV-1-associated disease. There is, however, a broad spectrum of diseases that are reported with infection other than HAM and ATL. Understanding the determinants of if, when, and what disease afflicts infected individuals needs to be further addressed. Created in BioRender.

HTLV-1 is primarily associated with the aggressive haematological malignancy ATL [1,2] and a chronic inflammatory disease called HTLV-1-associated myelopathies (HAM). The lifetime risk of developing ATL is estimated to be anywhere between 1 and 5% in carriers [91], and risks of HAM are estimated at a further 3% [92] (Figure 4). As the virus predominately affects host immune cells causing subclinical immune suppression, HTLV-1 infection is also linked with an elevated rate of opportunistic infections. This includes uveitis [93], Sjögren’s syndrome [94], infective dermatitis [95], bronchiectasis [96], and inflammatory disorders such as arthritis [97], amongst others [11,97,98,99]. Among HTLV-1 carriers, the risk of developing early neurological disorders can be as high as 24% [100,101]. Studies have shown that elevated PVL is the main risk factor for developing disease; however, more research is still required to illuminate the mechanisms governing disease development. There is an urgent need for expanded screening for HTLV-1 infection in asymptomatic carriers who can benefit from clinical monitoring. Clinically vulnerable patient populations in endemic areas who present with associated diseases such as Sjogren’s syndrome, thyroiditis, pulmonary disease, and opportunistic infections will benefit from anti-HTLV-1 antibody testing, and screening should be recommended in clinics for sexually transmitted infections.

## 2. In Vivo Assessment of Therapeutics

### 2.1. Cell Models

HTLV-1 was first isolated from ATL patient T cell lines and can infect a multitude of different cell types in vitro and in vivo. Since then, more ATL-derived cell lines have been isolated and cultured. As HTLV-1 has an affinity for cell-to-cell transmission, many HTLV-1-transformed cell lines have been developed, deriving from healthy leukocytes co-cultivated with leukaemic cells of ATL patients. HTLV-1 chronically infected cell lines have also been isolated from PBMCs of patients. HTLV-1 cell models have been an instrumental tool in understanding the key elements that play a role HTLV-1 pathogenesis and the testing of novel therapeutics. Importantly, many HTLV-1-infected cell models show an increase in spontaneous IL-2, which was later discovered to be constitutively expressed and induced by the tax and hbz genes [102]. Leukaemic cells in the majority of ATL patients were unresponsive to IL-2, suggesting that overtime proliferation outgrows its dependency of IL-2 in ATL patients. Generally, the relative mRNA expression of *tax* is reported to be higher than *hbz* in HTLV-1 chronically infected cell lines. However, the heterogeneity of HTLV-1 cell lines is highlighted in the absence of key viral genes, such as tax, which is not expressed by ATL-derived cell lines. Further characterisation of such expressions would provide an invaluable resource for in vitro antiviral screening. Commonly used HTLV-1 cell lines are listed in Table 4. 

An ongoing issue of HTLV-1-infected cell lines is the underrepresentation of more divergent subtypes, with recent studies suggesting that different HTLV-1 subtypes exert significantly different influences over the host immune system compared to the dominant cosmopolitan subtype [19]. The HTLV-1c subtype is prevalent in Australo-Melanesian regions and is the most sequence divergent of subtypes compared to the HTLV-1a cosmopolitan strain [117], with previous studies suggesting a distinct pathogenesis [96,118,119,120]. This highlights the need to develop therapeutics that have cross-subtype efficacy aided by HTLV-1c specific in vitro and in vivo models. To address this, a HTLV-1c chronically infected Jurkat T-cell line was created by co-culturing ex vivo HTLV-1c splenocytes harvested from the humanised mouse model developed at WEHI, with uninfected Jurkats. This T cell line expresses Tax, p19, env, and HBZ and is the first in vitro model of HTLV-1c infection. As most of our understanding and research in HTLV-1 is derived from the cosmopolitan subtype A, the development of a type C in vitro model will greatly help inform novel therapeutic approaches.

Early therapeutic screens can be performed in in vitro assays. For example, infection assays are often used to assess the capacity to inhibit HTLV-1 cell-to-cell transmission by the cocultivation of irradiated HTLV-1-donor cell lines to PBMCs from healthy individuals [121]. Enzymatic assays, such as the Amp-RT assay, were often used to assess HTLV-1 susceptibility to antiretrovirals such as reverse transcriptase, protease, or integrase inhibitors [122].

### 2.2. Animal Models

Over the past three decades, a variety of animal models have been developed to help elucidate the events of HTLV-1 pathogenesis and evaluate novel therapeutics. Multiple animal models are available, including mice, rats, rabbits, and non-human primates (NHPs). Other deltaretroviridae include the simian T-lymphotropic virus (STLV-1) and bovine leukaemia virus (BLV), both with large bodies of work useful for modelling HTLV-1 infection. A vaccine was recently developed for BLV, a disease which naturally infects cattle and shares common structural and functional genes with HTLV-1. The consequences of BLV vaccination will be explored further in its latter section. 

#### 2.2.1. Mice Models

HTLV-1 does not productively infect murine cells and must be first inoculated to establish persistent infection. The first HTLV-1 carrier mouse model was established in 1997 by intraperitoneal injection of MT-2 into C3H/HeJ and Balb/c strains of immunocompetent neonatal mice [123]. Three months post infection, HTLV-1 provirus was detected in PBMCs and lymphoid organs. Antibody responses against the Gag antigen was only observed in some Balb/c mice, with further studies confirming HTLV-1 persistence but the absence of antibody responses [124]. Since then, many immunocompetent HTLV-1 carrier mouse models have been developed. A broad spectrum of infected cell types can be observed in infected mice, including T cells and B cells, mimicking human infection. A caveat, however, is that immunocompetent HTLV-1 carrier mouse models demonstrate very little in vivo spread of infection with no evidence of disease, except for one report of tumorigenesis [125,126].

##### Xenograft Mouse Models for ATL

Xenografting involves the transplantation of human cancer cells into immunodeficient animals. Severe combined immunodeficiency (SCID) mice possess a mutation for the protein kinase, DNA-activated, catalytic polypeptide (PRKDC) gene, disabling VDJ recombination of B and T cell receptors and allowing them to be engrafted with human immune cells [127,128]. In 1992, SCID mice were used to successfully model ATL in mice [129], after treatment with anti-asialo GM-1 antibody to eliminate NK activity and inoculation with MT-2 cells. As SCID mouse models have been refined, successful engraftments in non-obese diabetic (NOD)/SCID mice with HTLV-1-transformed cell lines, ATL cells, and patient PBMCs have also been performed. In particular, engraftments with patient ATL cells have been shown to more accurately replicate disease [130,131]. NOD/SCID mice have been used as in vivo models for HTLV-1 induced ATL through engraftment with ATL-derived cell lines [132]. A proposed pre-clinical in vivo murine model of NOD/SCID mice injected ATL-patient-derived MET-1 cells was used to assess ATL therapies daclizumab, a mAb against IL-2Ra (CD25), combined with depsipeptide, an HDAV inhibitor. The study reports potent inhibition of tumour growth and prolonged mice survival, reflecting its efficacy in the treatment of ATL [133]. To further improve human cell transplant efficiency in SCID mice, the immune-deficient NOD/SCID mouse model was refined, generating NOD/Shi-scid IL2rγ^−/−^) mice (NOG). These mice carry the interleukin (IL)-2Rγ gene mutation and lack B- and T-cell development and NK cell function [134]. NOG mice inoculated with leukaemia cell lines and primary ATL cells expressing the tumour suppressor lung cancer 1 (TSLC1) gene experienced significantly higher tumour formation and aggressive infiltration in multiple organs.

##### Transgenic Mouse Models

Transgenic expression of Tax and HBZ proteins have been extensively used to model tumorigenesis facilitated by these two key players in various stages of HTLV-1 infection. Tax is a major oncoprotein in HTLV-1, being a transactivator of the HTLV-1 LTR but also playing roles in activating critical transcription factors [135], interference with cell cycle checkpoint control [136], and cellular transformation [137]. The first HTLV-1 Tax transgenic mice were developed in 1987, originally called HTLV-1 Tat, controlled by the LTR promoter [138]. These mice did not develop leukaemia/lymphoma but rather multicentric mesenchymal tumours, establishing Tax as an oncoprotein and HTLV-1 as a transformative virus. Since then, many Tax transgenic mice have been generated [139,140,141,142,143]. Tax expression in all transgenic mice leads to oncogenesis and other less typical manifestations of HTLV-1 infection; however, leukaemia/lymphoma induction is rare and does not fully capture the disease environment, but it has been useful to elucidate the role that tax plays in the disruption of T cell function and establish its role as an oncogene [138,144]. The traditional role assigned to Tax as a key player in leukaemia/lymphoma has also been challenged, as many studies report an absence of tax expression in ATL cells [87]. In 2006, an additional protein encoded by the antisense ORF was discovered. Hbz has been suggested to have a key role in leukaemogenesis and its maintenance, as it is constitutively expressed in all cases of HTLV-1-induced ATL. Transgenic expression of HBZ in CD4^+^ T cells induced leukaemia and lymphoma following a long latent period [145]. 

##### Humanised Mouse Models

ATL xenograft and transgenic mouse models have provided invaluable insight into immune response; however, they do not accurately capture the pathogenesis and persistence in HTLV-1 infection. Sub-lethally irradiated neonate mice are injected with human CD34^+^ umbilical cord stem cells (HUSCs) and subsequent reconstitution of a human immune system follows. The first humanised mice were used to model ATL after infection with irradiated MT-2 cells [146], the results of which revealed a similar profile to human HTLV-1 carriers. In particular, PVL was observed to increase with time, and HTLV-1 provirus was detected in primarily CD4^+^ but also CD8^+^ T cells. Tax gene suppression was observed in vivo; however, proliferation commenced upon in vitro culture. A more recent model details the intra-bone marrow injection (IBMI) of CD133^+^ cord blood cells into irradiated adult immunodeficient mice (IBMI-huNSG mice) to model ATL. Upon HTLV-1 infection, rapid CD4^+^ proliferation was detected in the periphery and clonal proliferation of CD25^+^ CD4 T cells [147]. ATL-like features and HTLV-1-specific adaptive immune responses were also observed in infected mice. HAM disease pathogenesis research has also benefited from humanised mouse models. A recent study engrafted CD34^+^ hematopoietic stem cells to Balb/c-Rag1-hu^−/−^ γc^−/−^ (Rag1) and Bone Marrow Liver Thymic (BLT) mouse models [148]. Following HTLV-1 infection, PVL was detected in peripheral blood two weeks post-infection, and at five weeks post-infection Tax was found to be significantly elevated in the spleen and CNS, peaking at 14 weeks post-infection and overrepresented in CD4^+^ T cells. The immune cell infiltration and resultant demyelination suggests humanised models can be infected with HTLV-1, resulting in viable infection. Whilst they do provide a more robust recapitulation of the disease microenvironment and immune responses; infection is short term, and the lack of a functional adaptive immune system does not capture the persistence of HTLV-1 infection [149].

Mouse models that are tailored to more divergent yet prevalent HTLV-1 subtypes are required to decipher the different underlying molecular mechanisms of infection, to understand virus biology, to identify preferred viral integration sites, and to interrogate which host genes are expressed at early and late stages of infection. Collectively, subtype-specific HTLV-1 mouse models will allow for more focussed discovery biology—i.e., why virus subtype HTLV-1c appears to preferentially elicit long-term disease complications that differ to HTLV-1a, including a higher risk of bronchiectasis—which in turn will also enable rationale therapeutic testing and design. To address this, we generated humanised mice by engraftment of CD34^+^ HUSC into NOD-SCID IL2Rgnull (NSG) that can be infected by intraperitoneal injection of lethally irradiated primary human PBMC from HTLV-1a or HTLV-1c-infected donors. These mice developed hallmarks of HTLV-1 disease including ATL and inflammation and enabled us to perform multi-omics approaches in comparison with human donor samples, which confirmed known but also identified novel underlying mechanisms of disease progression. Critically, this in vivo platform enables the testing of novel direct-acting antiviral treatments, as well as many of the novel therapeutic approaches outlined above that target HTLV-1 provirus or infection-related disease manifestations.

#### 2.2.2. Non-Human Primate Models

The first NHP infected with HTLV-1, reported in 1984, was established from rabbit lymphocytes co-cultivated with irradiated MT-2 cells [150]. The resulting Ra-1 cells were inoculated intravenously into Japanese monkeys. Seroconverted animals expressed HTLV-1 antigens and viral particles. Other NHPs susceptible to HTLV-1 infection include squirrel monkeys, cynomolgus monkeys, and rhesus macaques. In squirrel monkeys and cynomolgus monkeys, inoculation with MT-2 cells showed spontaneous expression of IL2 and HTLV-1 proteins in PBMCs [151]. HTLV-1-inoculated rhesus macaques developed arthritis, uveitis, and steroid-responsive polymyositis [152]. Squirrel monkeys have also been used to assess the efficacy of early HTLV-1 vaccine development [153]. NHPs indeed recapitulate most aspects of viral infection, replication, and pathogenesis; however, the high cost of research and regulations underpin its scarce use in experimental research.

#### 2.2.3. Other Animal Models

Other models for HTLV-1 infection include rabbits and mice. Rabbits are an early researched model for HTLV-1, and this was first established in 1984 when the inoculation of rabbits with Ra-1 cells returned HTLV-1 seropositivity [154]. Rabbits inoculated with HTLV-1 do not develop HTLV-1-associated diseases; however, they present with persistent HTLV-1 infection. The New Zealand White rabbit model was used to determine the transformation timepoint of HTLV-1 tropism [155]. Here, HTLV-1 was detected in both CD8^+^ and CD4^+^ T cells one-week post-infection (wpi); at five wpi, the predominant cell type that was observed was CD4^+^ T cells, indicating that preferential tropism arises in the chronic phase of the disease. HTLV-1 transgenic flies, Drosophila melanogaster, expressing Tax and Hbz were proposed to study Tax-driven oncogenesis in vivo [156,157]. Tax was shown to modulate the expression of NF-κB and the enhancer Polycomb Repressive Complex 2 (PRC2), which governs persistence and cellular transformation. Overexpression of Hbz was observed to prevent Tax-induced NF-κB and PRC2 activation, shedding light on the co-modulatory interplay of Tax and Hbz [157]. 

## 3. Current Treatments

Despite the early discovery and remarkable genetic stability of HTLV-1, specific and effective anti-HTLV-1 treatments and vaccines have yet to be developed. The advent of Antiretroviral Therapy(ART) revolutionised retroviral treatment strategies, transforming the Human Immunodeficiency Virus (HIV) into a manageable, chronic disease [158]. ART has ushered about a paradigm shift in the approach to retroviral treatment, yet, contrary to the HIV field, such success has not been imitated in the treatment of HTLV-1 [159,160,161]. This section discusses current interventions, with a focus on the limitations of ART in the treatment of HTLV-1 and its associated diseases.

### 3.1. Antiretroviral Therapies and Limitations for HTLV-1

The mechanisms of ARTs involve targeting distinct stages of the viral life cycle, including interference with viral entry, reverse transcriptase inhibitors, DNA integrase inhibitors, and protease inhibitors. The immediate goal of ART is to reduce the viral load below levels detectable by assays (50 RNA/mL) and to elicit the eventual normalisation of CD4^+^ T cell count (>500 cells/µL) [162,163]. ART targets viral entry, life cycle, and replication but fail to eliminate HTLV-1 reservoirs due to overlooking their clonal expansion replication. This differs from HIV’s virion-based replication, leading to lower ART efficacy against HTLV-1, explored further in this section. The cessation of therapy in HIV is associated with a strong viral rebound to pre-therapeutic plasma viral loads [164,165,166] and may be similarly mimicked in the incidence of successful treatment of HTLV-1 with ART.

#### 3.1.1. HTLV-1 Entry Inhibitors

Whilst HTLV-1 relies on different cell membrane receptors to facilitate entry compared to HIV, SU and TM subunits of the envelope are common to both viruses. There are no approved therapeutic agents targeting the HTLV-1 entry process, despite there being many HIV-1 inhibitors that are FDA approved. However, previous studies have characterised the potential of synthetic peptides which interfere with conformational changes in TM subunits, inhibiting env-mediated membrane fusion and HTLV-1 entry. In 2008, Mirsaliotis et al. reported that leash-like synthetic peptides mimicking the C-terminal α-helical domain of TM are potent antagonists of membrane fusion and viral entry [167,168]. The inhibitory effects of heparin against HSPGs are well characterised against several retroviruses [169,170,171,172,173]. Jinno-Oue et al. reported that chondroitin sulphate type E (CSE) and heparin exhibited anti-HTLV-1 activity by interacting with env proteins at the C-terminal of SU to inhibit viral entry to a human T cell line (MOLT-4) [174]. 

#### 3.1.2. Nucleoside Reverse Transcriptase Inhibitors (NRTIs) and Non-Nucleoside Reverse Transcriptase Inhibitors (NNRTIs)

The current approach to targeting RT in HTLV-1 is based on the successes of HIV-1 RT enzymatic inhibitors, being nucleoside RT inhibitors (NRTIs) and non-nucleoside RT inhibitors (NNRTIs). NRTIs, being nucleoside analogues, are competitive inhibitors of the RT catalytic site, interfering with endogenous dNTP binding and preventing the polymerisation process [175]. NNRTIs are non-competitive inhibitors of RT, binding instead at an allosteric pocket and altering the structural conformation of the binding site [176]. Many NRTIs and NNRTIs are FDA approved for the treatment of HIV-1; however, to date, there are no HTLV-1 specific inhibitors nor any published crystal structures for HTLV-1 RT that allow for more precise characterisation. Amino acid sequence alignment of HTLV-1 and HIV-1 RT has shown that the two RTs share ~25% sequence identity and ~45% sequence similarity [73]. Despite the presence of common conserved retroviral motifs, the residues of the HIV-1 NNRTI binding pocket differ vastly from HTLV-1, nullifying the potential to repurpose HIV-1 RT NNRTIs for HTLV-1 treatment.

Few HIV-1 NRTIs inhibit viral replication of HTLV-1 in single and combination therapy, despite the multitude of those that are FDA approved. The pyrimidine nucleoside analogue zidovudine AZT, repurposed from successful early HIV therapy [177], was explored as an early therapy. AZT was shown to inhibit HTLV-1 infection in PBMCs, co-cultured with HTLV-1-infected MT-2 cells in graduated concentrations, as well as significantly inhibit cell proliferation [178,179]. The acyclic nucleoside phosphonate tenofovir has also demonstrated long-term inhibition of HTLV-1 infection in PBMCs in concentrations as low as 0.1 µM [180]. Both reverse transcriptase inhibitors, AZT and Tenofovir, have been shown to block primary infection and reduce proviral load in NOD-SCID, Common γ-Chain Knockout Mice [181]. However, this protection was only no longer conferred one week post infection due to the dominance of clonal proliferation, and viral titres rapidly bounced to untreated levels after one week of administration. Similarly, the susceptibility of HTLV-1 to the potential anti-HIV-1 NRTIs Zdv, zalcitabine (ddC), didanosine (ddI), Lamivudine (3TC), and stavudine (d4T) for HTLV-1 was assessed by Garcia-Lerma et al. in 2001, showing resistance to 3TC. HTLV-1 resistance to 3TC was shortly confirmed by multiple in vitro assays [182], and the antiviral potency was determined to be more than 100 times lower than AZT, discounting its potential for clinical relevancy. In 2006, a randomised, double blind, placebo-controlled clinical trial observed the combination therapy potential of zidovudine (AZT) plus lamivudine (3TC) in the treatment of HAM. After 24 weeks, there was no significant reduction in PVL in PBMCs or changes in clinical measures [161]. Contrastingly, other studies have suggested that the use of NRTIs may potentially activate the tumour suppressor gene p53 in healthy cells, inhibiting telomerase to cause cell death [183,184,185].

#### 3.1.3. Integrase (IN) Inhibitors

The development and clinical applications of integrase (IN) inhibitors, like the antiretrovirals discussed previously, have been catalysed by the race to attenuate HIV infection. IN is proposed to be a promising target for anti-HTLV-1 therapeutic development, as some structural properties of IN are common between HTLV-1 and the already established HIV-1 [186], and IN has no human orthog. The two main classes of HIV-1 IN inhibitors, styrylquinolines (SQLs) and diketo acids (DKAs), act at different points of the integration process. A study in 2008 examined the effects of SQLs and pre-established HIV-1 IN-targeted DKAs in an in vitro strand-transfer assay and ex vivo infection of PBMCs with lethally irradiated HTLV-1-positive MT2 cells [187]. The compounds which were active in vitro reduced cell proliferation ex vivo at lower concentrations and resulted in a dramatic decrease in number of migration events and PVL in early infection. Despite the prospect of IN inhibitors as an anti-HTLV-1 treatment option, it may prove futile when addressing the oversaturated population of people chronically infected with HTLV-1. Nevertheless, initial HTLV-1 infection is characterised by horizontal viral replication prior to the dominative onset of clonal expansion of infected cells, so IN inhibitors may assist in attenuating early infection. The first IN inhibitor approved for HIV-1 clinical use is Raltegravir. Studies have characterised its effects in treating HTLV-1, with reports of a transient reduction in PVL in patients [160] and inhibition of transmission and immortalisation in isolated human PBMCs [188]. Ultimately, neither study observed significant changes in HTLV-1 PVL beyond the course of therapy.

#### 3.1.4. Protease Inhibitors (Ritonavir)

HTLV-1 protease (PR) is critical for the maturation step during viral replication by processing the viral polyproteins Gag and Gap-Pro-Pol. Several PR inhibitors are FDA approved and clinically used in the treatment of HIV-1 and acquired immune deficiency syndrome (AIDS). The crystal structure of HTLV-1 PR was recently characterised [189] (Protein data bank code 3WSJ) and bore highly similar three-dimensional folding to HIV-1 PR, despite only sharing 26% amino acid sequence identity and 38% amino acid sequence similarity [73]. The most notable of the FDA-approved PR inhibitors to be applied in the treatment of HTLV-1 is ritonavir, which did display anti-leukaemic activity against ATL cells ex vivo; however, this was mostly due to the inhibitory effects exerted on the NF-κB target rather than PR [190]. This will be discussed further in Section 2.2.

### 3.2. Treatment of HTLV-1 Associated Diseases 

The three primary diseases associated with HTLV-1 are ATL, HAM, and the onset of inflammatory syndromes. The mechanisms that govern the likelihood of which ailment manifests are poorly understood. Given the absence of approved HTLV-1 antiviral therapeutics, the majority of readily available drugs only treat the symptoms of disease.

#### 3.2.1. ATL

Approximately 5% of HTLV-1 patients are at risk of developing ATL, an aggressive mature T cell malignancy [104,191]. Despite the revolution in molecular anti-cancer therapies in the 21st century, the prognosis of ATL remains poor [192]. ATL therapeutic strategies for patients are determined largely by clinical subtype (acute, lymphoma, chronic, and smouldering) [12,193]. Some countries also adopt differing treatment regimens, as not all therapeutics of ATL are universally available. Aggressive forms of ATL are treated with intensive chemotherapy, coupled with concurrent or low-dose azidothymidine, an NRTI developed for HIV-AIDS, and interferon-alpha (AZT/IFN-α) for maintenance [194]. Where possible, therapeutic providers are encouraged to consider early upfront allogenic haemopoietic stem cell transplants (allo-HSCT) for all eligible patients [194,195,196]. AZT/IFN-α is not currently approved for ATL treatment in Japan, despite being the worldwide standard for acute, chronic, smouldering symptomatic and PCT-ATL subtypes [194]. This is possibly due to outcomes of aggressive ATL treated with AZT-IFNa remaining poor and frequently being associated with opportunistic infections [194]. As a result, several newer agents have been trailed in Japan, most notably the monoclonal anti-CCR4 antibody (mAb) mogamulizumab, which has been approved to treat upfront and refractory ATL [197]. Mogamulizumab targets CCR4, a chemokine receptor highly expressed by HTLV-1-infected T cells [198,199,200], with phase I and II clinical trials resulting in an overall response rate (ORR) of 50% [201,202,203]. In 2015, a subsequent randomised phase II study was conducted to compare the ORR and complete response rate (%CR) of mogamulizumab to mLSG15, a dose-intensified chemotherapy [204]. This study reported a higher %CR in patients who received combination therapy compared to chemotherapy alone (52% vs. 33%, respectively); however, opportunistic infections were frequented in the combination arm, including a cytomegalovirus infection observed in 14% of participants. Mogamulizumab administered to patients prior to allo-HSCT is associated with an increased risk of grade 3 to 4 acute graft-versus-host disease (GVHD)-related mortality, with poor clinical outcome within 50 days of allo-HSCT [205]. Therefore, a 50-day washout period is recommended for patients eligible for mogamulizumab and allo-HSCT. Another potential mAb investigated for HTLV-1 treatment is anti-transferrin receptor antibodies, notably A24. HTLV-1-infected cells constitutively express high levels of surface transferrin receptor [206], which A24 is directed against. A pre-clinical study characterised the potential of A24, which induced apoptosis and blocked ex vivo proliferation of malignant T cells from both chronic and acutely infected patients [207]. Lenalidomide is a thalidomide analogue approved for the treatment of multiple myeloma, mantle cell lymphoma, and myelodysplastic syndrome [208]. It is approved in Japan for the treatment of relapsed and refractory (R/R) ATL with improved ORR [209]. A recent case report outlined promising clinical results of low-dose lenalidomide, following chemotherapy and mogamulizumab for maintenance treatment [210], which warrants further investigation in combination approaches for recently approved therapeutics.

In recent years, the EZH1–EZH2 dual inhibitor valemetostat has been shown to suppress HTLV-1-infected cell proliferation, reduce tumour size, and potentially improve outcomes of R/R ATL in clinical trials. EZH1/2 are two isoforms of the enhancer of zeste and are alternative subunits of Polycomb Repressive Complex 2 (PRC2), a chromatin-modifying enzyme causing epigenetic modifications, such as H3K27me3 upregulation, and linked with oncogenesis [211,212]. In 2022, phase 2 clinical trials assessed the efficacy of 200 mg oral valemetostat in 25 participants with a median of three prior lines of therapy [213]. Treatment with valemetostat resulted in 48% ORR and manageable adverse events in over 20% of participants. Valemostat was approved in Japan in 2022 for the treatment of ATL; however, it also harbours potential for HAM treatment, as it has been demonstrated to inhibit the proliferation of HTLV-1-infected cell lines derived from patients with HAM [214]. The clinical potential of treatments targeting epigenetic markers warrants further exploration.

Tax has been extensively researched in its pathogenic potential and is thus an attractive therapeutic target. It is associated with T cell transformation, resulting in the activation and proliferation of infected cells and HTLV-1 leukaemogenesis [46,68,215,216]. Tax-targeted therapies have been examined to a lesser degree. ST1926 is a synthetic retinoid that was reported to repress Tax expression and inhibit infected cell proliferation in ATL lines by inducing apoptosis through upregulation of p53 [217]. Cyclosporin A, an immunosuppressant that inhibits T cell activation and cytokine production, was observed to inhibit Tax expression in HTLV-1-infected cells and the nuclear expression of tax-related transfer factors ATF-1/2 [218]. Interestingly, niclosamide, which is an anti-helminthic drug approved for the treatment of tapeworms, downregulated Tax and pro-survival Bcl-2 proteins to induce apoptosis of HTLV-1-transformed cells [219]. Notably, the degradation of the Tax protein in the proteasome was observed and subsequently downregulated 5′ viral gene transcription of HTLV-1 [219]. As Tax is the main target of cytotoxic lymphocytes [220,221], studies have suggested that cells with silenced Tax expression are preferentially selected during disease progression [80,222]. Despite driving initial viral sense transcription from the 5′LTR, Tax protein is mostly undetectable in chronic infection due to subsequent methylations and proviral deletions elicited by HTLV-1 immune escape mechanisms [223,224]. Tax is a desirable therapeutic target at early stages of HTLV-1 infection and oncogenesis and warrants further investigation into its anti-ATL potential, especially coupled with existing and approved agents.

Another potential therapeutic target in the treatment of HTLV-1 is NF-κB, as its pathway is chronically activated in HTLV-1-transformed cell lines, even in the absence of tax expression [225]. Histone deacetylase inhibitors (HDACIs) are anti-cancer agents that prevent the reactivation of transcriptionally suppressed genes. The anti-leukaemic potential of HDACIs (including valproic acid, vorinostat, romidepsin, panobinostat, and entinostat) has been characterised by multiple in vitro studies in HTLV-1-transformed and ATL-derived cell lines by blocking the Notch pathway to decrease NF-κB and induces apoptosis [226,227]. Other investigated inhibitors include Bay 11-7082 and indole-3-carbinol [228], both inducing apoptosis in HTLV-1-infected T cell lines, illuminating the potential for NF-κB as an anti-leukaemic agent. 

ATL is a devastating disease. A variety of novel therapeutics have been developed to trounce this potential consequence of HTLV-1; however, individually, their overall impact on patient outcomes have proven limited. Learning from the advances in HIV-1 therapeutics, where combination treatment has led to a marked improvement in patient outcomes, a similar approach may need to be developed. This challenge can be best surmounted by an international collaborative effort.

#### 3.2.2. HAM

HAM is a chronic neuroinflammatory disease, with most patients presenting with a slow deterioration in mobility and bladder function [229]. Most patients will require walking aids within a decade of manifestation, with many becoming wheelchair-dependent a decade later [230]. Regardless of clinical progression, the PVL of HAM patients remains high throughout infection [230]. Although the relationship between HTLV-1 and HAM has been characterised for many decades, little progress has been made in its treatment. Current approaches that comprise the backbone of clinical management can be classed as disease-modifying treatments (DMTs) or symptomatic therapy. Symptomatic management is helpful in improving patient mobility and quality of life; however, they fail to alter the course and progression of disease [231]. HAM can be categorised into “subtypes” based on the speed of disease progression. The International Retrovirology Association’s guidelines for the management of HTLV-1-associated myelopathy/tropical spastic paraparesis (2018) recommends differing DMT regimens for rapid, slow, or non-progressing subtypes. The most ideal DMT would be the eradication of HTLV-1-infected cells; however, no effective antiviral therapeutics have been developed to date. Most DMTs aim to suppress the immune response, modulate inflammation, and reduce the PVL to modify the course of disease [231]. The three most characterised classes of DMTs for HAM are antiretrovirals, corticosteroids, and IFN-a [232].

Antiretrovirals targeting HTLV-1 work to suppress PVL in HAM and were detailed previously in ATL treatments, the therapeutic potential of which is replicated in HAM intervention [161,233]. The lack of efficacy of ARTs in treating HAM is a consequence of the support that clonal proliferation brings to chronic HTLV-1 infections and the absence of active replication and de novo infection. 

Outside of this, corticosteroids are the most common treatment for patients in all stages of HAM disease, and its effectiveness in improving motor function has been reported in numerous observational studies [234,235,236,237,238]. Patients with a short duration of disease and high inflammatory activity have been reported to benefit the most from corticosteroids [239,240,241]. Until recently, no studies have evaluated their clinical efficacy with randomised control trials. In 2021, Yamauchi et al. conducted the first randomised, double-blinded, placebo-controlled trial including 8 rapidly and 30 slowly progressing HAM patients [242]. Rapid progressors were assigned a 3-day course of intravenous methylprednisolone with oral prednisolone, while slow progressors received oral prednisolone or placebo. The primary outcomes measured improvements in motor and gait function using the Osame Motor Disability Score (OMDS) and 10 m walking time (10 mWT) for rapid progressors and 10 mWT baseline changes for slow progressors. Three out of four rapid progressors achieved the primary outcome, although no significant difference was recorded for the slow progressing group. Well-characterised, serious complications of corticosteroids are secondary infections, including urinary tract infections compounded by neurogenic bladder issues caused by HAM [243], and these were replicated in both randomised control trial groups. This study indicates that corticosteroid therapy may be beneficial for rapid progressing HAM, and larger-scale studies will be necessary to provide more insight into its therapeutic potential.

IFN-α is an immunosuppressant that has also been studied in a randomised controlled trial setting. A total of 48 HAM patients were separated into groups and treated with different IFN-α concentrations for four weeks, and clinical evaluations measured motor dysfunction, urinary disturbances, and changes in neurologic signs [244]. The therapeutic benefits of IFN-α increased with higher concentrations. Despite the favourable clinical effects, its long-term utility is challenged by a high probability of adverse reactions [245]. Further studies monitoring the long-term benefits of IFN-α and measuring patient response through cerebrospinal fluid (CSF) and PVL markers may shed light on its potential. IFN-β1a was also researched in an uncontrolled “proof of concept” trial, although no significant clinical progression was recorded [246].

The calcineurin inhibitor, cyclosporin A, has been used as a steroid-sparing therapy and was demonstrated to decrease HTLV-1 PVL and show clinical improvement in HAM patients. Notably a proof-of-concept study was conducted in 2012, which reported that cyclosporin A reversed clinical deterioration in early-phase HAM patients [247]. A case study in 2015 supported these claims, with clinical improvement in motor function and decreased PVL [248]. However, the safety profile of cyclosporin A remains unclear and should be further investigated.

mAbs in the treatment of HAM have been researched to a lesser degree. The first molecular, targeted mAb specific for HAM was against the Humanized IL-2 receptor α-chain antibody (anti-Tac or daclizumab), which was found to lower PVL in PBMCs with selective downregulation of HTLV-1-infected CD4^+^ T cells [249]. However, significant improvements to patient outcome were not observed in the nine patients treated. Discussed previously for the treatment of ATL is the humanised anti-CCR4 mAb mogamulizumab. A phase I/IIa clinical trial involving 21 patients also reported its utility for HAM [203]. Mogamulizumab reduced HTLV-1 PVL, cytokine production, and spontaneous proliferation, improved motor ability in 32% of patients, and reduced spasticity in 79% of patients. The most notable side effects were rashes, with the serious adverse events lymphopenia and leukopenia reported in 33% of patients each. Recently, a multicentre, randomised phase III study of mogamulizumab was completed to verify its efficacy in a larger population [250]. Participants received 0.3 mg/kg intravenous mogamulizumab over a 24-week double-blind period, a placebo-controlled period, a 24-week open-label period, and an extension treatment period. At the end of the double-blinded period, no significant difference was observed in the primary (OMDS) or secondary endpoints assessing motor function (10 WT). However, a significant decrease was observed in PVL, CSF CXCL10/neopterin, the significance of which is discussed in Section 3.4. A higher incidence of rash was reported (69.2%); otherwise, the safety profile was unchanged, as with clinical benefit. Despite this, the significant decrease in inflammatory markers proves that mogamulizumab is a promising therapeutic to pursue.

Other therapeutics investigated in the treatment of HAM include danazol, valproic acid, ascorbic acid, and plasmapheresis. Danazol is an anabolic steroid with a safer toxicity profile than prednisone. Limited studies have evaluated its therapeutic potential, with reports of improvement in patient mobility and decreases in spasticity [251,252]. Mild liver toxicities were reported in higher danazol dosages. An open-label trial was conducted in 2011 examining the effects of valproic acid, a lysine deacetylase inhibitor that modulates gene expression [253]. PVL and lymphoproliferation were not significantly altered by therapeutic intervention, and only three out of nineteen participants reported a significant increase in motor function. In addition, the consistent advent of adverse effects including drowsiness and tremor may be poorly tolerated. An open-label trial investigated the efficacy of vitamin C therapy (ascorbic acid) in seven HAM patients [254]. Participants were given a daily oral dose (35–40 mg/kg) for three to five successive days, followed by a two-day withdrawal period. Patients demonstrated significant improvements in motor function but reduced with treatment cessation. CSF HTLV-1 antibody titre remained unchanged throughout treatment. This study suggests a favourable immunomodulatory action of high-dosage ascorbic acid in HAM patients, and its antiproliferative effects have been corroborated in a recent in vitro study [255]. The effects of ascorbic acid can be confirmed with further research involving a larger and more varied cohort and the inclusions of PVL measurements and modern clinical biomarkers. Plasmapheresis was demonstrated to improve gait and reduce sensory and sphincter disturbances in 11 out of 18 patients with 4–6 sessions in a two-week period [256]. Furthermore, a lower serum HTLV-1 antibody titre was observed, suggesting that plasmapheresis may offer a temporary improvement in HAM patients. 

A currently active trial in HAM therapeutics examines the therapeutic potential of teriflunomide, a drug used to treat multiple sclerosis. Teriflunomide was demonstrated to inhibit abnormal CD4^+^/CD8^+^ T cell proliferation associated with HTLV-1 infection, coupled with a significant decrease in PVL without affecting cell viability [257]. In 2021, phase I/II clinical trials were initiated examining the therapeutic potential of teriflunomide in HAM patients (ID: NCT04799288). Patients will receive 14 mg oral dosage daily for nine months with a subsequent three-month follow-up period. The primary outcome measure is ex vivo spontaneous lymphoproliferation, with secondary outcomes measuring immune activation patterns and PVL of PBMCs and CSF. 

HAM is a complex disease with enormous gaps in available therapeutics that halt or reverse patient deterioration. The management of pathological consequences is a temporary and unviable solution to a chronic and persisting disease, and the diversity of current HAM interventions reflects the absence of effective pharmaceutics. Other interventions not discussed in this section include clinical trials on the efficacy of green tea [258] and fermented milk [259]. The primary challenges in HAM treatment are current gaps in the understanding of disease pathogenesis and patient enrolment for clinical trials. Addressing this gap involves the standardisation of disease categorisation and methods to evaluate improvement, including clinical indicators that correlate with disease progression.

#### 3.2.3. Other Inflammatory Diseases

HTLV-1 infection is also associated with a range of other inflammatory diseases and infections. Previous sections have outlined the lack of specific and targeted treatments for HTLV-1 inflammatory and immune related conditions, which is mirrored in the treatment of other associated inflammatory diseases and secondary infections. Well-characterised HTLV-1-associated diseases and infections include, but are not limited to, uveitis [93], Hashimoto’s thyroiditis and Grave’s disease [98], pulmonary diseases [17], inflective dermatitis [95], inflammatory myositis [260], and allergy inflammatory disorders [261]. The percentage or likelihood of infected patients developing additional HTLV-1-associated diseases is not well understood. Data are scarce on the prevalence of secondary infections, with previously conducted studies being observational in nature and limited by their small cohorts and geographic restraints. Table 5 summarises HTLV-1-associated diseases and their treatments. Lesser-known medical burdens of HTLV-1 infection extend beyond this list, as patients report experiencing non-specific neuropathic/non-neuropathic pain alongside infection [262].

### 3.3. HTLV-1 Pre-Exposure Prophylaxis (PrEP)

HIV Pre-Exposure Prophylaxis (HIV-PrEP) is effective in reducing the likelihood of HIV acquisition in non-HIV-infected people at a high risk of exposure. The current FDA-approved PrEP is an oral combination of the NRTIs emtricitabine (FTC) and tenofovir or intramuscular injections of long-acting integrase strand transfer inhibitor (INSTI) [269]. As detailed in previous sections, these antiretrovirals may be of little benefit to chronically infected HTLV-1 patients. However, as HIV-PrEP acts through early inhibition of replication, similar therapeutic strategies may be repurposed to prevent early HTLV-1 transmission. Despite this, there are currently no studies that have evaluated the plausibility of HTLV-PrEP [270].

### 3.4. Biomarkers for Disease

As detailed in previous sections, the absence of predictors for disease progression in HTLV-1 infection is an unaddressed gap in the current literature. Establishing clinically useful biomarkers will prove instrumental in revolutionising patient care and clinical research. Current studies suggest that the risk of developing ATL or HAM is not random. The manifestation of disease may be sequence-independent in HTLV-1; thus, the host response to the virus is a more reliable indicator for disease prediction. There is strong evidence suggesting that increased HTLV-1 PVL in PBMCs is a potent risk factor for disease progression [216,271,272,273]. Previous studies have reported that symptomatic patients have significantly higher HTLV-1 PVL compared to asymptomatic controls [120,273,274,275]. However, measuring PVL in PBMCs is both costly and time-consuming. To enable wider testing, the simpler and more accessible and cost-effective whole-blood PVL measurement can be explored to facilitate broader testing and early detection of HTLV-1. Other potential biomarkers for HAM are CSF inflammatory markers, which examine the levels of CXCL10 [241] and neopterin [239]. Current biomarker candidates are mostly immunogenetic in nature, summarised in Table 6. The human leukocyte antigen (HLA) is crucial in immune response induction, as well as the PVL of HTLV-1. HTLV-1 viral protein p12 inhibits HLA-1 molecules on infected cell surfaces to mediate immune escape. Specific HLA alleles have been reported as determinants of HAM and ATL development and disease severity [276,277], but allele determinants present in one population may not be reflected in another. Specific polymorphisms in cytokine genes have also been reported as a potential determinant for disease [278]. The HTLV-1 functional genes tax and hbz are also associated with the development of disease, as outlined previously. Studies have previously reported that elevated expression of tax mRNA in PBMCs washigher in patients presenting with HAM compared to asymptomatic controls [279]. These findings have also been compared to hbz mRNA expression [85], where hbz mRNA expression levels were decreased after IFN-α treatment and clinical improvement, suggesting its utility as a treatment response biomarker.

## 4. Future of HTLV-1 Treatments

Many treatments thus far for HTLV-1 have been repurposed from other diseases, so the current state of therapeutics will benefit from novel approaches. Addressing the biggest roadblock in treatment, the provirus, which governs viral persistence and reactivation, is critical when developing an anti-HTLV-1 therapeutic. Novel therapeutic approaches, specifically targeted genetic and molecular therapies, have been widely explored in the treatment of HIV-1, and similar therapeutic approaches can be adapted for HTLV-1, a virus comparable in its genealogy and aetiology. These therapeutic approaches that can be paralleled between HIV-1 and HTLV-1 include Zinc Finger Nucleases (ZFNs), Crispr/Cas9/12/13 gene editing, and RNA interference (RNAi). HTLV-1 is a perfect candidate for gene therapy with its limited sequence diversity, high conservation across subtypes, and its two identical LTRs, which act as two possibly highly potent target sites. The focus of this review will be on the advances and limitations in small interfering RNA (siRNA)-directed RNAi and its potential as an anti-HTLV-1 therapeutic. This follows precedent set by the success of the mRNA COVID-19 vaccines and the five FDA-approved siRNA therapeutics and further highlights the potential for antiviral RNA-based therapeutics. 

### 4.1. Gene Editing

ZFNs act as a pair of synthetic endonucleases, introducing a double-stranded break (DSB) onto a cognate DNA site, with the resulting cleavage set to be repaired through nonhomologous end joining (NHEJ). The NHEJ pathway is often inaccurate, and resulting localised insertions and deletions act as a sufficient method of inactivating the gene [288]. ZFNs are highly specific to the target site and have been widely adapted in the treatment of HIV [289,290,291,292], with several ongoing studies (NCT03617198, 02500849). In 2013, Tanaka et al. developed ZFNs that targeted the HTLV-1 LTR regions and examined their potential in HTLV-1-transformed and ATL cell lines [293]. These studies demonstrated potent disruption of the HTLV-1 LTR promoter and subsequent proviral gene expression, anti-tumour effects in vivo, and, most importantly, apoptosis of HTLV-1-infected cells triggered by DSBs in the LTR. Despite the inhibition of infected cell proliferation, there remains a subset of infected memory cells in chronic patients that do not actively express viral mRNA. ZFN technology is potent and specific; however, surface markers specific to HTLV-1-transformed and -untransformed cells remain the missing link necessary for translating gene editing to a viable therapeutic.

CRISPR (clustered regularly interspersed short palindromic repeat) is a genome-editing technique mediated by endonucleases, most commonly Cas9, and guides RNAs (gRNAs) to introduce DSBs into the gene target [294]. The CRISPR/Cas9 editing approach has demonstrated some success in targeting HIV-1 in vitro and in vivo [295,296], the most promising of which is a proof-of-concept study observing effective elimination of proviral DNA in infected mice [297], but this result has yet to be replicated in HTLV-1. Recent research has speculated on the success of CRISPR editing of HTLV-1, with some proposing that HTLV-1 is an excellent model for gene editing [298]. The majority of discourse surrounds the introduction of breaks along the *tax* and *hbz* viral genes, interrupting immortalisation and suppressing infected cell growth and survival. As HTLV-1 is highly genetically conserved within hosts and among isolates, it serves as a powerful candidate for focused gRNA targeting and generalised gene manipulation approaches. The successful recognition and disruption of the promoter via ZFNs provides additional support for the feasibility of CRISPR editing. Common in many gene-editing approaches, however, is the potential for off-target mutagenesis and issues with delivery. A persisting probability in CRISPR/Cas9 systems is the introduction of changes in unrelated genes due to the relatively high mismatch tolerance of gRNAs [299]. Cas9 is also a large effector nuclease, being 160 kDa in size, and effective delivery poses a challenge [300]. Smaller effector nucleases have been researched as an alternative to Cas9, including Cas9, Cas12a (Cpf1), Cas13, and Cas14 (a/b/c) [301]. CRISPR-directed gene editing can potentially be a powerful method of targeting HTLV-1 genes, with *tax* and *hbz* being genetically conserved across subtypes A and C (93% for tax and 85% for hbz, respectively); however, there are many hurdles to overcome before it can be considered a clinically viable alternative.

### 4.2. Gene Silencing by RNA Interference (RNAi)

Gene silencing induced by RNA interference (RNAi) has been successfully adapted to silence the HIV-1 proviral genome and similarly has potential in the treatment of HTLV-1. RNAi uses short, complementary RNA sequences (19–23 nucleotides) to mediate sequence-specific silencing of a target promoter region. The molecules needed to induce RNAi-silencing pathways include short interfering RNA (siRNA), short hairpin RNA (shRNA), or microRNA (miRNA) [302]. All three molecules share a common mode of action—the specificity of base pairing interactions with a gene target—although the focus of this review will be on the therapeutic potential of siRNAs.

#### 4.2.1. RNAi Pathways

**Figure 5:**
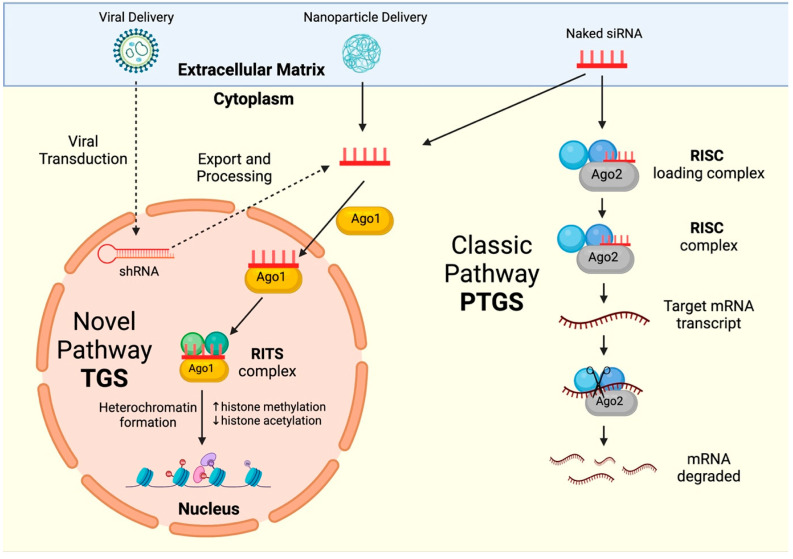
Mechanisms of siRNA-induced gene silencing. Both RNAi pathways can exert genetic repression by viral and non-viral mechanisms of delivery. PTGS mostly occurs in the cytoplasm as the RISC machinery initiates specific mRNA cleavage. TGS is localised to the nucleus and is mediated by the RITS complex, acting through repressive epigenetic modifications. Ago1, Argonaute 1; Ago2, Argonaute 2; shRNA, short hairpin RNA; RISC, RNA-induced silencing complex; RITS, RNA-induced transcriptional silencing complex; TRBP, transactivating response (TAR) RNA-binding protein; TNRC6, trinucleotide repeat containing 6 protein faily. Created in BioRender.

RNAi-silencing pathways can be largely grouped into two categories: the classic RNAi pathway, otherwise known as post-transcriptional gene silencing (PTGS), and the novel RNAi pathway, termed transcriptional gene silencing (TGS) or epigenetic silencing. The PTGS pathway is initiated via a siRNA complementary to a cytoplasmic mRNA target. The siRNA binds to an Argonaute protein (Ago2), forming the RNA-induced silencing complex (RISC), which degrades the complementary mRNA. The novel gene-silencing pathway is localised to the nucleus and drives silencing via transcriptional repression at the gene promoter. Argonaute 1 (Ago1) protein ushers the promoter–complementary siRNA to enter the nucleus and forms the RNA-induced transcriptional silencing (RITS) complex, which induces heritable, epigenetic modifications that repress transcription, thereby silencing gene expression [303] (Figure 5). Idiosyncratically, the classic pathway has been researched to a far greater extent than the novel pathway; however, its noninheritable nature poses a drawback, as a continuous supply of siRNA is required to maintain a therapeutic effect. This was addressed using viral delivery methods to provide long-term siRNA expression, but this solution does not come without its caveats, as the oversaturation of cellular machinery may pose risks of toxicity and off-target effects [304,305,306]. The LTR promoters of HTLV-1 are primary regions of interest for silencing. As HTLV-1 has an exceptionally conserved genome, it is a promising candidate for the sequence-specific and highly potent RNAi.

#### 4.2.2. Controlling Chronic Viral Infections

Gene therapy approaches to controlling chronic viral infections such as HIV-1 aim to suppress latency in retroviral infections as opposed to transient viral repression. Core therapeutics for HIV-1 are more advanced than HTLV-1 and can help inform an approach for functional therapeutic development. An eradication approach in the treatment of latently infected HIV-1 cells is known as the “shock and kill” technique, which aims to reactivate infected cells by “shocking” them using latency-reversing agents (LRAs), although this strategy is accompanied by significant challenges. LRAs are commonly investigated for the treatment of HIV-1 and exert their action by activating viral transcription for infected cells and then “kill” reservoir cells through cytopathic mechanisms, the host immune response, or other targeted means. There are several caveats intrinsic to directing LRAs against HIV-1, as it acts through the reversal of epigenetic silencing signatures present in the HIV-1 promoter in latent phases. This can result in the global activation of any promoter carrying the targeted epigenetic marks and is not always specific to the HIV-1 promoter [307]. Another challenge in the “shock and kill approach” with LRAs is the lack of reproducibility across different cell models, a result that has also been observed clinically [308]. The successful reactivation and subsequent suppression in vitro have failed to produce impacts on the latent reservoir for patients in vivo [308]. 

Another approach to the treatment of the latent reservoir is the “block and lock” strategy, which specifically induces TGS in the viral promoter to incite targeted suppression of replication, thereby blocking transcription to lock the promoter in a state of latency. The first anti-HIV-1, siPromA, was first identified in 2005 [309], and targeted the unique and highly conserved NK-κB transcription factor sites in the HIV-1 promoter to induce potent epigenetic silencing [70,310,311,312]. Due to the highly conserved nature of the NK-κB binding sequence, there are no shared commonalities with the human genome, and targeting of this site has not revealed any identifiable off-target effects [313]. Recently, the efficacy of siPromA in the treatment of HIV-1 has been characterised by in vivo humanised mouse models, demonstrating robust protection against infection by lowering the HIV-1 cell-associated RNA levels in siPromA-expressing CD4^+^ T cells isolated from blood and lymphoid tissue [70,314]. siRNAs can also be multiplex to provide more robust antiviral protection by broad-spectrum coverage across multiple subtypes [315,316]. Further advances in this field harbour considerable implications for the future of developing a functional cure for similar viruses, such as HTLV-1. 

Amongst the diverse family of nucleic-acid-based therapeutics considered for antiviral treatment are antisense oligonucleotides (ASOs). ASOs are single-stranded DNA oligonucleotides around 12–25 bp in length [317]. Like siRNAs, ASOs act through complementary base pair binding, targeting disease transcripts to cause mRNA degradation or sterically prevent binding with effector proteins. There are eight ASOs that have been FDA approved for clinical use. The first FDA-approved ASO, vitravene (Fomiversen), in 1998, treated CMV retinitis in immunocompromised AIDS patients [318]. By 2006, Fomiversen was revoked in the United States and the European Union (EMA). The second ASO to be FDA approved in 2013 was Mipomersen for the treatment of familial hypercholesterolemia. Mipomerson was not approved by the EMA due to hepatic safety concerns [319]. Following its approval, a further six ASO therapies were approved for various treatments, four of which were for the treatment of Duchenne muscular dystrophy (DMD), a rapidly progressing neuromuscular disorder [320]. Despite the extensive research and development of ASOs that have led to the numerous clinically approved therapies, there are still many limitations to its technology. The biggest challenge is delivery and cellular uptake, like most nucleic-acid-based therapeutics (discussed in more depth in the following section). There are no approved antiviral ASOs; however, the COVID-19 pandemic has instigated exploring its potential for the treatment of SARS-CoV-2 [321]. Due to successful early studies on the development of siRNA therapeutics targeting HIV-1, ASOs have also been explored as a new therapeutic modality, albeit with limited capacity. Early studies have chemically modified an ASO (g-ASs) that can inhibit reverse transcription on a variety of RNA sequences, including HIV-1 [322]. Recent advancements in chemical modification have seen the development of an anti-HIV-1 ASO (FANA ASO), which suppresses replication in human PBMCs [323]. 

#### 4.2.3. Challenges in RNAi for HTLV-1

**Figure 6:**
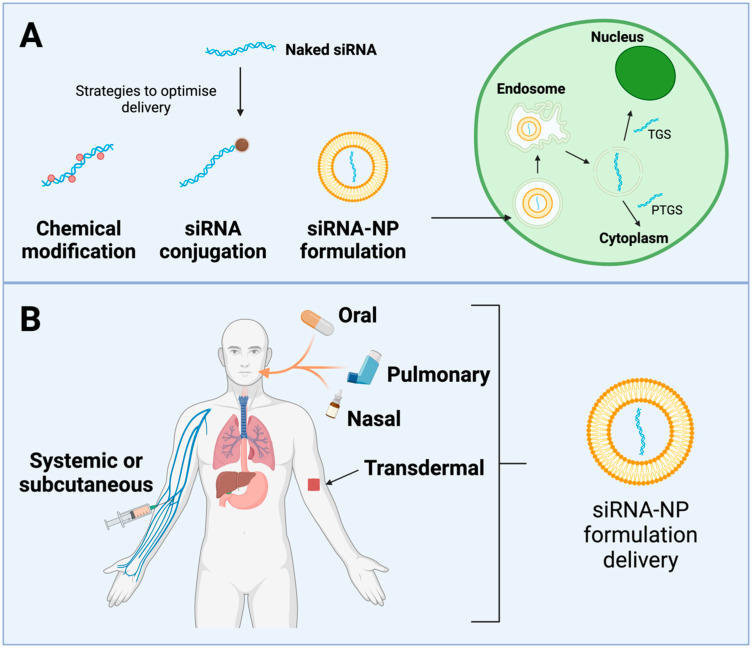
Routes of administration for nanoparticle delivery of RNAi therapeutics. (**A**) Chemical modifications, siRNA conjugations, and nanoparticle formulations can be used to improve the outcomes of delivery. Nanoparticle formulation helps to facilitate cell entry and endosomal escape to yield enhanced epigenetic silencing. (**B**) The different routes of administration for nanoparticle formulation. Created in BioRender.

The ambitious strategy proposed by novel siRNA therapies does not come without its controversies, with efficient delivery and off-target effects being a common hurdle for any type of gene therapy. With various in silico programmes available to define siRNA sequences, there is a distinct lack of in vitro and in vivo studies conducted in HTLV-1. Addressing the challenges surrounding the epigenetic silencing of HIV-1 may provide a model for those of HTLV-1 due to their genetic similarity. Delivering genetic modifications through siRNAs presents a major technical challenge in this field; the most common routes of delivery include the systemic delivery of naked siRNA, lentiviral vectors, and alternative non-viral methods based on nanotechnology [302]. Each of these methods presents their own caveats, including systemic delivery being vulnerable to enzymatic degradation, hepatic elimination, and rapid renal filtration [324,325]. Whilst lentivirus vectors are most commonly used, safety and potential deleterious effects due to the integration site are widely contended. An alternate delivery method is utilising nanocarriers; however, challenges include correct biodistribution, cell entry, and endosomal escape. Bypassing each of these issues could possibly be achieved through chemical modification of siRNAs, including conjugation with macromolecules and nanoparticle formulations [326,327] (Figure 6). 

A precedent for nanocarriers was set by the first FDA-approved siRNA therapeutic, Partisiran, which is formulated with a lipid nanoparticle encasing [328]. The advantage offered by a nanoparticle formulation is the ease of cell entry and endosomal escape, thereby leading to more efficacious gene silencing. Despite this, nanoparticle delivery methods still do not address the issue that comes with clearance, as even with intravenously administered siRNAs, typical hepatic accumulation limits access to target sites [325,329,330]. Another possible mode of delivery is macromolecule conjugation, utilised by Givosiran, whereby the therapeutic siRNA was conjugated to N-acetyl-d-galactosamine tris domains (GalNAc) [331]. However, some reports have documented that these formulations show cytotoxicity and the activation of innate immune responses with limited metabolic stability [332]. Other siRNAs orchestrating hepatic degradation include Lumasiran [333], another GalNAc–ligand conjugated siRNA; Inclisiran [334]; and, recently, Vutrisitan [335]. Excitingly, preclinical studies for an siRNA developed with a biomimetic nanoparticle formulation against Chronic Hepatitis B is currently underway [336]. If successful, this would be the first antiviral siRNA to reach FDA approval, setting the precedent for other viral-targeted siRNAs. Being one of the biggest roadblocks to the clinical implementation of RNAi therapeutics, effective delivery must be prioritised in the early stages of drug development.

Another innovative strategy lies in the possibility of extracellular vesicles (EVs), specifically exosomes, as a delivery platform. Exosomes are small nanoparticles (50–100 nM), released from actively replicating cells that are taken up by neighbouring cells. Exosomes have been used as both a therapeutic delivery vehicle [337] and diagnostic tool for a range of applications, including MS, HD, and cancers [338,339]. Recently, anti-HIV-1 therapeutic exosomes were engineered to package and deliver promoter-targeted zinc finger proteins (ZPAMt) to induce the “block and lock” of HIV-1-infected cells, including cells in the brain [340]. Effective silencing of HIV-1 was also observed with repeated doses of exosomes (100 × 10^9^) in 25 g mice with no observed hepatotoxicity. After cessation of ZPAMt expression, long-term epigenetic silencing was observed. Recently, a similar anti-HBZ ZFP was developed and observed to reduce the proliferation and viability of patient-derived ATL cell line TL-Om1 [341]. Notably, similar to ZPAMt, this anti-HBZ ZFP can also be packaged into exosomes for systemic delivery [342]. Lastly, exosomes can be generated to target cells in a receptor directed manner, and when targeted to the Spike protein also inhibit SARS-CoV2 infection [343]. Exploring exosomes as next-generation delivery platform for the epigenetic modulation of HTLV-1 may prove a safe, highly innovative approach to treating infection and ameliorating disease.

### 4.3. The BLV Vaccine: A Model for HTLV-1 Immunisation and Vaccination

Bovine leukaemia virus (BLV) is a deltaretrovirus that causes a common neoplastic disease in cattle called enzootic bovine leukosis (EBL), characterised by B-cell leukaemia/lymphoma. The disease is responsible for significant economic losses in the dairy industry, estimated to exceed USD 525 million annually in the United States alone [344]. BLV shares common retroviral structural proteins Gag, Pol, and Env, as well as Tax and Rex in HTLV-1. Tax is a common viral oncoprotein to both viruses, and it is thought to induce malignant progression following infection with BLV [345]. Upon integration, BLV also displays low levels of transcription, with 5% of animals infected developing leukaemia/lymphoma [346,347,348]. Similarly, the 5′LTR transcriptional promoter selectively represses and activates upon viral expression to facilitate immune escape, demonstrated by a decrease in PVL upon increasing promoter efficiency [349]. By defining the epigenetic factors involved in viral repression and expression, BLV offers a valuable model for the assessment and genesis of novel antiviral gene target therapies. Recently, an attenuated BLV vaccine was developed that harbours a mutation in the env gene and deletions of sense and anti-sense accessory genes (AS1-S, AS1-L, R3 and G4) [350]. The vaccine was delivered in a dairy herd in Argentina and conferred immunity in 28 out of 29 cows over a 4-year period. The vaccine was shown to be safe and effective, with low PVL and no pathogenic events recorded after administration. The success of the BLV vaccine was attributed to its high genomic stability and administration around the expiry of protection offered by the maternal colostrum. Following previous failed attempts, including DNA vaccines that did not prevent later infection due to transient activation, including recombinant vaccinia virus (RVV) and inactivated BLV, the attenuated BLV succeeded with deletions in genes that regulate infectivity and replication [351]. The BLV vaccine may provide a model for HTLV-1 vaccination, with the need for the stimulation of humoral and cytotoxic immune responses. Both tax and hbz should be considered important gene targets, as they are increasingly implicated in persistence and disease induction. Such a vaccine for HTLV-1 could be distributed to at-risk populations in endemic regions.

## 5. Conclusions

With no therapeutic strategy or standardised patient care addressing HTLV-1 infection, there is an urgent need for antiviral treatments and vaccines. The genetically conserved 5′ and 3′LTR regions of HTLV-1 offer promising candidates for epigenetic silencing. Along with the novelty of siRNA therapeutics, there are still many challenges posed for the literature to address, including efficient and targeted delivery and clinical translation. The recent development of the BLV vaccine also provides a useful model for potential HTLV-1 vaccination. Despite these challenges, the development of RNAi therapeutics has illuminated an exciting new modality of therapy and could have considerable implications for the future of treating HTLV-1.

## Figures and Tables

**Table 3:** Comparison of HTLV-1 and HIV-1 infection.

Property	HTLV-1	HIV-1
Infectious Duration	Lifelong	Lifelong
Primary Immune Targets	CD4^+^/CD8^+^ T cells	CD4^+^ T cells
Transmission	Cell-to-Cell	Virus particles
Viral Expression	Low	High
Immune consequences	Overactive inflammation	Immune deficiency
Treatment	Few targeting symptoms	ART targeting virus
Tumorigenesis	Direct	Indirect

The differences in viral propagation and survival inherit to HTLV-1 and HIV-1 necessitates different approaches in their treatment. This is discussed in further detail below in Section 2.

**Table 4:** Commonly used HTLV-1 cell lines.

	Cell Line	Cell Type	IL-2 Dependency	Antigen Expression
HTLV-1-transformed cell lines	MJ [103]	Lymphoblast	Independent	CD2^+^; CD3^+^; CD4^+^
	MT-2 [104]	Lymphoblast	Independent	CD4^+^; CD25^+^; FoxP3^+^
	MT-4 [105]	Lymphoblast	Independent	CD4^+^
	HuT-102 [1]	Cutaneous T Lymphocyte	Dependent	CD4^+^
	C81-66 [106]	Lymphoblast	Independent	CD4^+^
HTLV-1 chronically infected cell lines	C91/PL [103,107]	Umbilical cord-blood T cells	Independent	CD4^+^
	MS-9 [108]	Cord-blood T cells	Dependent	CD4^+^
ATL cell lines	MT-1 [109]	Lymphoblast	Independent	CD4^+^; Tax^−^
	MT-3 [110]	Lymphoblast	Independent	CD4^+^; Tax^−^
	ATL-2 [111]	Lymphoblast	Independent	CD4^+^; CD3^−^
	TL-Om1 [112]	Lymphoblast	Independent	CD4^+^; Tax^−^
	F6T [113]	Lymphoblast	Independent	CD4^+^; CD25^+^
	K3T [113]	Lymphoblast	Independent	CD4^+^; CD25^+^
	S1T [113]	Lymphoblast	Independent	CD4^+^; CD25^+^
	ATL-T [114]	Lymphoblast	Independent	CD4^+^
	ATL-35T [115]	Lymphoblast	Independent	CD4^+^
	ATL-55T [116]	Lymphoblast	Dependent	CD4^+^
	Su9T01 [113]	Lymphoblast	Independent	CD4^+^

**Table 5:** Treatments for HTLV-1-associated diseases and infection.

Disease	Treatment
HTLV-1-associated uveitis	Topical or systemic corticosteroids and mydriatics [263]
HTLV-1-associated Sjögren’s Syndrome	Artificial lubricants such as saliva/tears replacement solutions coupled with systemic pharmacotherapy with pilocarpine hydrochloride [264]
HTLV-1-associated Hashimoto’s thyroiditis and Graves’ disease	Partial or complete thyroidectomy and hormone replacement therapy [264]
HTLV-1-associated infective dermatitis	Antibiotics and corticosteroids [264]
HTLV-1-associated pulmonary disease	Long-term corticosteroid therapy [265]
HTLV-1-associated inflammatory myositis	Currently no available therapeutic; myositis is resistant to corticosteroids and immunomodulatory therapies such as cyclosporin [266,267]
HTLV-1-associated arthritis	Immunomodulatory therapies such as TNF-*α* [268]
HTLV-1-associated conjunctivitis sicca syndrome and interstitial keratitis	Artificial lubricants such as tears, cyclosporine eye drops [264]

Relapse is common when treatment is halted. Other less-common inflammatory conditions reported with HTLV-1 carrier status include tubulointerstitial nephritis, mixed connective tissue disease, and idiopathic autoimmune hepatitis. Also reported are non-communicable diseases like CKD and diabetes.

**Table 6:** Gene polymorphisms associated with HTLV-1 disease.

Gene	Allele/Genotype/Haplotype	Disease	Indication
HLA	DRB1*DQB1* 0101, 1502, 0803 [280]	HAM	Increased risk
	DRB1*DQB1* 1501, 0901, 1401 [280]	ATL	Increased risk
	A*24 and Cw*01 [280]	ATL	Protective
	Cw*7, B*7 and DR1 [280]	HAM	Increased risk
	A*02 [281]	HAM ATL	Protective Increased risk
	*HLA*-B*5401 [276]	HAM	Increased risk
	A*03 and DQB1*0501 [282]	ATL	Protective
	B*15 and B*53 [282]	ATL	Increased risk
IFN-*γ*	*IFNG*+874A/T [283]	-	High PVL
IL-18	−607CC [284]	-	Protective
	−607AC [284]	-	Increased risk
TNF-*α*	TNFA−857C/T [285]	ATL	Increased risk
IL-10	*IL10*−592A/C [286]	HAM	Increased risk High PVL
	*IL10*−819*C/T and −592*C/A [287]	-	Increased risk

Factors that determine the increased risk of HTLV-1 infection and genesis of HAM and ATL are poorly understood. The complicated relationship between genetic factors and HTLV-1 susceptibility, whilst recognised, would undoubtedly benefit from further research.

## Data Availability

Not applicable.
